# Investigation of Staphylococcus strains with heterogeneous resistance to glycopeptides in a Turkish university hospital

**DOI:** 10.1186/1471-2334-5-31

**Published:** 2005-05-05

**Authors:** Yasar Nakipoglu, Sengul Derbentli, Atahan A Cagatay, Handan Katranci

**Affiliations:** 1 Department of Microbiology and Clinical Microbiology, Faculty of Medicine, University of Istanbul, Istanbul, Turkey; 2 Department of Infectious Diseases and Clinical Microbiology, Faculty of Medicine, University of Istanbul, Istanbul, Turkey

## Abstract

**Background:**

The hetero-glycopeptide intermediate staphylococci is considered to be the precursor of glycopeptide intermediate staphylococci especially vancomycin intermediate *Staphylococcus aureus *(VISA). For this purpose, we aimed to investigate the heterogeneous resistance to glycopeptide and their frequencies in 135 *Staphylococcus *strains.

**Methods:**

Heterogeneous resistance of *Staphylococcus *strains was detected by inoculating the strains onto Brain Heart Infusion agar supplemented with 4 mg/L of vancomycin (BHA-V4). Agar dilution method was used for determining MICs of glycopeptides and population analysis profile was performed for detecting frequency of heterogeneous resistance for the parents of selected strains on BHA-4.

**Results:**

Eight (6%) out of 135 *Staphylococcus *strains were exhibited heterogeneous resistance to at least one glycopeptide. One (1.2%) out of 81 *S. aureus *was found intermediate resistance to teicoplanin (MIC 16 mg/L). Other seven strains were *Staphylococcus haemolyticus *(13%) out of 54 coagulase negative staphylococci (CoNS). Six of the seven strains were detected heterogeneously reducing susceptibility to vancomycin (MICs ranged between 5–8 mg/L) and teicoplanin (MICs ranged between 32–64 mg/L), and one *S. haemolyticus *was found heterogeneous resistance to teicoplanin (MIC 32 mg/L). Frequencies of heterogeneous resistance were measured being one in 10^6 ^– 10^7 ^cfu/ml. MICs of vancomycin and teicoplanin for hetero-staphylococci were determined as 2–6 folds and 3–16 folds higher than their parents, respectively. These strains were isolated from six patients (7%) and two (4%) of health care wokers hands. Hetero-VISA strain was not detected.

**Conclusion:**

Heterogeneous resistance to glycopeptide in CoNS strains was observed to be significantly more emergent than those of *S. aureus *strains (vancomycin P 0.001, teicoplanin, P 0.007). The increase MICs of glycopeptide resistance for subpopulations of staphylococci comparing with their parents could be an important clue for recognizing the early steps in the appearance of VISA strains. We suggested to screen clinical *S. aureus *and CoNS strains, systematically, for the presence of heterogeneously resistance to glycopeptide.

## Background

Since the first report published from Japan in 1997 on vancomycin-intermediate (MIC 8–16 mg/L) *Staphylococcus aureus *(VISA)[1] and studies from different countries, suggested that the emergence of glycopeptide resistance among staphylococci is a global issue [2]. Survey of resistance to glycopeptide indicates that there are two types of resistances. The first type is homogeneous resistance which occurs as a result of thickening in the cell wall, such as being in VISA strain [3]. The other homogeneous resistance was reported from Michigan in June 2002 [4] in which MIC of vancomycin for the *S. aureus *strain was found ≥ 32 mg/L and regarded as the first vancomycin resistance *S. aureus *(VRSA). VRSA strain has vanA gene of enterococci which explains the origin of VRSA strain [5].

The main difference between the VISA and VRSA strains is that the later could transfer the glycopeptide resistance among the strains by plasmids. Homogeneous resistance could be determined by conventional methods as described by the National Committee for Clinical Laboratory Standards (NCCLS) [6]. The second type is heterogeneous resistance, which was described firstly by Hiramatsu et al [1] from Japan in 1997, and it is represented with strain Mu3 (hetero-VISA). Mu3 strain has been isolated from an old man with pneumonia who did not give any response to vancomycin therapy, although MIC of Mu3 was 4 mg/L it has been containing a mutant subpopulation of cells having intermediate resistance to vancomycin (MIC 8 mg/L).

It is difficult to detect heteroresistance by NCCLS methods because the concentration of bacterial population which was advised by NCCLS (5 × 10^5 ^cfu /mL) is not sufficient to detect heteroresistance (1 in 10^6 ^cfu/mL). Population analysis method is the most common screening method for the detection of heteroresistant staphylococci [1]. Depending on many studies hetero-VISA is precursor strain for VISA [7]. A growing concern over recent reports from many countries suggests that the hetero-VISA is prevalent and responsible for the failure of vancomycin therapy [5]. Clonal spread of these strains via hands of Health Care Workers (HCW) has also been reported [8]. Beacuse of low virulance of CoNS, they have been dismissed as being culture contaminants in the past, however, in recent years they have been assumed of greater importance as true pathogens and the emergence of strains with decreased levels of susceptibility to vancomycin and teicoplanin have been noticed in many studies reviewed by Cercenado et al [9]. Istanbul University hospital is a large hospital with 1500 bed capacity, we aimed to determine heterogeneously resistance to vancomycin and teicoplanin in *S. aureus *and CoNS strains for the first time in Turkey.

## Methods

### Strains

A nonrepetitive 135 *Staphylococcus *strains were collected from September 2001 to April 2002 from the Faculty of Medicine of the Department of Microbiology and Clinical Microbiology of the University of Istanbul in Turkey. These were 87 strains (73 *S. aureus *and 14 CoNS) which were clinical or colonized isolates of the skin of different body sites of the hospitalized patients, and 48 strains (40 CoNS and 8 *S. aureus*) colonized on the skin of the hands of HCWs. CoNS strains were identified to the species level by standard biochemical procedures [10]. The species distribution of CoNS were as follows: *S. haemolyticus *18 (33.3%), *S. epidermidis *10 (18.5%), *S. simulans *6 (11.1%), *S. schleiferi *6 (11.1%), *S. lugdunensis *3 (5.5%), *S. arlettae *3 (5.5%), *S. xylosus *3 (5.5%), *S. hominis *3 (5.5%), *S. capitis *1 (1.8 %), and *S. warneri *1 (1.8%). *Staphylococcus aureus *ATCC 29213 was used as control strain for determining MICs of glycopeptides. Heterogeneous VISA strain Mu3 was used as control in population analysis profile method.

### MICs criteria of Glycopeptides

MICs were evaluated according to the NCCLS breakpoints for *Staphylococcus *strains [11] and were as follows: For vancomycin ≤ 4 mg/L as susceptible, 8 – 16 mg/L as intermediate, and ≥ 32 mg/L as resistance. For teicoplanin: ≤ 8 mg/L as susceptible, 16 mg/L as intermediate and ≥ 32 mg/L as resistance.

### Detection of *Staphylococcus *strains with hetero-reduced susceptibility to vancomycin

This method was applied as described previously by Hiramatsu et al [1]. Briefly, overnight cultures were adjusted to 0.5 McFarland turbidity and 10 μl of the suspension was inoculated onto Brain Heart Infusion agar (BHA) (BBL; Becton Dickinson and Company, Cockeyvilles, USA) plates containing 4 mg/L of vancomycin (Sigma, chemical Inco, Germany) (BHA-V4), and incubated at 37°C for 48 hours. If a countable number (one to 30) of colonies grew, the strain was considered to have potential heteroresistance to glycopeptide and was confirmed with population analysis profile method. The strain was accepted as susceptible to vancomycin if an obvious growth was not seen on the inoculated plate.

### Population analysis profile method

This method was used for detecting of staphylococci subclones intermediate or resistant to glycopeptide. For this purpose, the parents of the selected colonies on BHA-V4 were used and the method was performed as previously defined by Hiramatsu et al [1]. A 50-μl aliquot of the overnight isolates and control strain (Mu3) in Brain Heart Infusion (BHI) broth were adjusted to optical density of 578 (10^8 ^cfu/ml) and serial 10-fold dilutions were spread over two different BHA plates, one containing vancomycin at concentrations ranging from 1 to 10 mg/L (ten dilutions), and the other containing teicoplanin (Gruppo Lepetit S.P.A, Italy) at concentrations ranging from 1 to 64 mg/L (two fold dilutions). After incubation at 37°C for 48 hours, the number of viable cells were calculated and plotted on a semi-logarithmic scale. Heterogeneous Resistant Staphylococci was defined as any screen-positive strain which contained subpopulations with MIC > 4 mg/L for vancomycin or ≥ 16 μg/ml for teicoplanin at a frequency of 1 in 10^6 ^cfu/mL or higher.

### Detection MICs of vancomycin and teicoplanin

MICs were determined for the parents of strains grown on BHA-V4 by using cation-adjusted MHA (Oxoid, Unipath LTD, Hampshire, England) and two fold increments of vancomycin at concentrations ranging from 0.25–32 mg/L and teicoplanin at concentrations ranging from 0.25–128 mg/L and the agar dilution method was performed according to the NCCLS [6].

### Patients Medical Record

Records of patients who had colonized or infected with *Staphylococcus *strains with decreased susceptibility to glycopeptides were reviewed and data related to the patients age, gender, ward, underlying disease, specimen, duration of hospitalization and previous treatment with glycopeptides were evaluated.

### Statistical analysis

Fisher's test was used for comparing of glycopeptide resistance between *S. aureus *and CoNS strains.

## Results

### Detection of staphylococci with reduced susceptibility to glycopeptides

Nine (7%) out of 135 *Staphylococcus *strains grew on BHA-V4 screening plates. Two of these strains were found as being methicillin susceptible *S. aureus *(MSSA, 4502 and 4503) and other seven strains were found as being methicillin resistant CoNS (MRCoNS) (strains from 4504 to 4510).

### MICs of vancomycin and teicoplanin of strains grew on BHA-V4

MICs of vancomycin and teicoplanin for the parents of subclones were as follows: for each of 4502 and 4503 strains were found being susceptible (1 mg/L), for seven MRCoNS strains were found susceptible to vancomycin and ranged between 1–4 mg/L. MICs of teicoplanin were found being intermediately susceptible (16 mg/L) for two MRCoNS (4508, 4510) strains and susceptible (1 to 8 mg/L) for another five strains.

#### Population analysis profile

**Figure 1:**
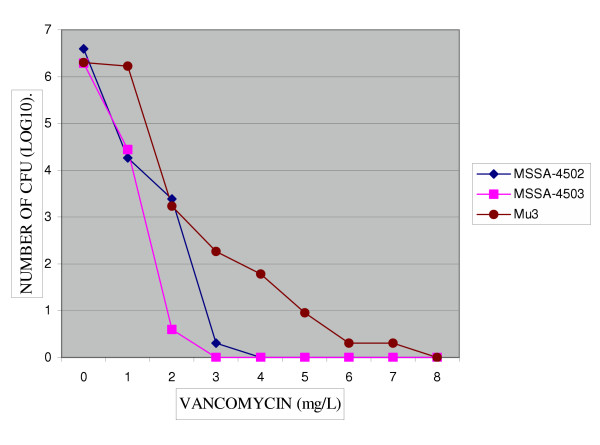
Population analysis of two *S. aureus *strains (4502,4503) grew on BHA-V4. MICs of vancomycin for subclones were susceptible (3–4 mg/L).

**Figure 2:**
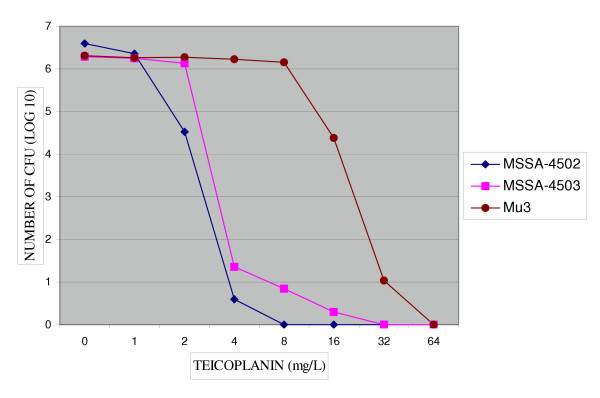
Population analysis of two *S. aureus *strains grew on BHA-V4. MICs of teicoplanin for subclones of MSSA-4502 were susceptible (8 mg/L) and for MSSA-4503 were resistant (32 mg/L).

**Figure 3:**
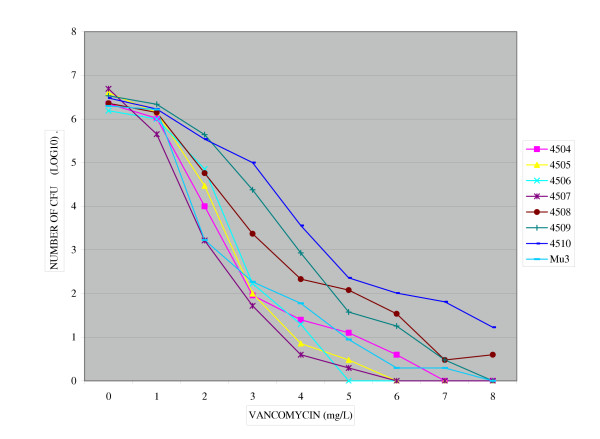
Population analysis of MRCoNS strains grew on BHA-V4. MICs of vancomycin for six MRCoNS strains subclones (4504, 4505, 4507, 4508,4509,4510) were reduced (5–8 mg/L) and for one strain (4506) subclones were susceptible (4 mg/L).

**Figure 4:**
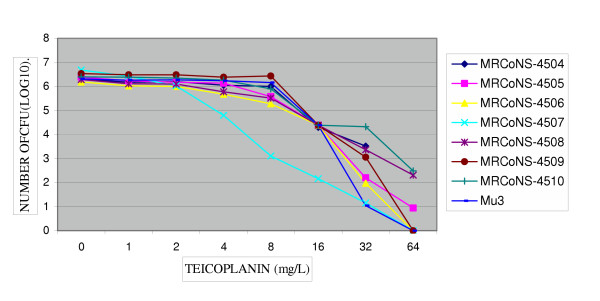
Population analysis of seven MRCoNS strains (4504–4510) grew on BHA-V4. MICs of teicoplanin for these strains subclones were resistant (32–64 mg/L).

Although *S. aureus *no 4502 grew on BHA-4, its sublones were susceptible to vancomycin (MIC 4 mg/L) and teicoplanin (MIC 8 mg/L). This strain was not accepted as heterogeneous strain, whereas those of *S. aureus *4503 were susceptible to vancomycin (MIC 3 mg/L) and resistant to teicoplanin (MIC 32 mg/L) and the strain was hetero-teicoplanin resistant *S. aureus *(TRSA). MICs for subclones of seven MRCoNS (all were *S. haemolyticus*) of vancomycin (except strain 4506: MIC 4 mg/L) were reduced susceptibility and varied between 5–8 mg/L, and all were resistant to teicoplanin (MICs were ranged between 32–64 mg/L). Heterogeneous frequencies in staphylococci strains were observed 1 in 10^6 ^– 10^7^cfu/ml (Fig. 1, 2, 3, 4). Therefore, heteroresistance to glycopeptide antibiotics was found to be one (1.2%) out of 81 *S. aureus *strains and seven (13%) out of 54 CoNS strains. Heteroresistance to glycopeptide was higher in *S. haemolyticus *(39%) and it was found as being the only species among CoNS that has showed heteroresistance to glycopeptide.

#### Glycopeptide resistance in clinical and colonized strains

Six (7%) out of 87 patients and two (4%) out of 48 HCWs hands isolates displayed heterogeneous resistance to at least one glycopeptides. Only one strain (4503) out of eight *S. aureus *strains colonizing HCWs hand's skin was hetero-intermediate resistance to teicoplanin, and none of the 73 screened clinical *S. aureus *strains had reduced susceptibilty to glycopeptides. For CoNS strains, six out of 14 patients isolates and one out of 40 HCWs hand skin strains were hetero-resistant to vancomycin and teicoplanin with the exception of 4506 strain; it was susceptible to vancomycin and resistant to teicoplanin (Table 1).

**Table 1:** Clinical features of patients infected or colonized with MRCoNS with reduced susceptibility to glycopeptides

Patients Strain No	Age/sex	Ward	Clinical feature	Underlying disease	Specimen	Duration of hospitalization (days)	Previous treatment with glycopeptide (days)	Treatment with glycopeptide (days)	Clinical outcome
4504	46/F	Infectious Diseases	Pneumonia	Trauma	tracheal aspiration fluid	20	No	vancomycin (14)	Improved
4505	33/M	Haematology	Septicemia	CML	Groin skin	27	No	No	NT
4506	44/M	Haematology	Septicemia	AML	Abdominal wall skin	46	vancomycin (20)	No	NT
4507	46/F	Haematology	Septicemia	ALL	Axilla skin	60	vancomycin (10)	No	NT
4508	26/M	SICU	Septicemia	Trauma and Fracures Of mandibula and maxilla	Blood	31	No	vancomycin (10)	Improved
4510	75/F	Infectious Diseases	Septicemia	Surgical operation of Colon	Blood	6	No	vancomycin (14)	Death not related to staphylococcal infection

Abbreviations: M, male; F, female; CML, chronic myelogenous leukemia; AML, acute myelogenous leukemia; AML, acute lymphocytic leukemia; SICU, surgical intensive care unit; NT, staphylococci skin colonization not treated.

## Discussion

Heterogeneous resistance to teicoplanin among *S. aureus *strains was very low (1.2%), whereas this resistance among MRCoNS was higher (13%). Using vancomycin was licensed in 1994 in Turkey and preceded teicoplanin about two years. From this point of view, the expectation was to detect *Staphylococcus *strains with vancomycin resistance more than teicoplanin resistance. But our results have shown that seven *S. haemolyticus *were resistant to teicoplanin (MIC ≥ 32 mg/L) and one *S. aureus *strain has an intermediate resistance to teicoplanin (MIC 16 mg/L), whereas resistance to vancomycin in the same eight strains subclones was increased mildly above the susceptible range (4 m/L) in six strains, and intermediate range (8 mg/L) in two strains. It is hard to link between the glycopeptide use and appearance of resistance to this antibiotic group in our strains, because except in two strains (4506 and 4507), no previous treatment with any glycopeptide was found through viewing the medical reports of the patients colonized or infected with these strains. On the other hand, the MICs of vancomycin even for these two strains were 4 mg/L for the former and 5 mg/L for late strain. The selective pressure, independently from the treatment with glycopeptide, could be the major factor in the heterogeneous resistance among strains (Table 1).

Historically, *S. aureus *acquired teicoplanin resistance before it acquired vancomycin resistance. Teicoplanin resistance is frequently accompanied with a small increase in vancomycin resistance [12]. Shlaes et al [13], demonstrated that PBP2 is overproduced in a TRSA mutant strain (MIC 16 mg/L) compared with its parent strain. Over-production of PBP2 is also observed in the hetero-VISA strain Mu3 which is resistant to teicoplanin. Most of the studies on glycopeptide resistance mechanisms were done with *S. aureus *strains and it may be also true for CoNS strains; but, more studies are needed.

Species variation has been detected in vancomycin susceptibility of CoNS, according to many studies [14-16]. This resistance was more common in *S. haemolyticus *and *S. epidermidis*. In this study, we also obtained similar results, in which *S. haemolyticus *was the only species revealed with high resistance (39%) to glycopeptide among ten different species. Indeed there is not a sufficient explanation which relates directly the glycopeptide resistance and species phenomenon.

A survey of hetero-VISA revealed differences in the prevalence of hetero-VISA between the countries and even between university and nonuniversity hospitals in the same country. In Japan after the first report on hetero-VISA in 1997, Hiramatsu et al [1] have reported a result of hetero-VISA as 3%, however, no hetero-VISA strain was found in a study conducted in the same year by Ike et al [17] on 6,625 *S. aureus *of university and nonuniversity hospitals strains. Prevalence of heteroVISA strains in the other European countries like Italy, Germany, France, Netherland were reported as 1.1%, 0.21%, 0.6%, 6%, respectively, [18-21] and in Thailand and Korea as 1.93% and 0.54%, respectively [22,23]. We did not isolate heteroVISA strain, but we detected heteroTRSA. Heterogeneous resistances to vancomycin and teicoplanin among CoNS were found to be 11% and 13%, respectively, and higher than those obtained by Gruneberg et al [24], a European collaborative study on 1594 CoNS from bloodstream infections, and they found that all of the isolates were susceptible to vancomycin and only 0.7% of them were resistant to teicoplanin.

Examination of six heteroMRCoNS showed that three strains colonized the skin of different body sites of immunocompromised patients and three others were isolated from clinical specimens (Table 1). The MICs of vancomycin for these strains were varied between the susceptible and intermediate ranges, whereas teicoplanin was in the resistance ranges. The strain no 4509 which was isolated from the hand skin of HCW was intermediate resistant to vancomycin (7 mg/L) and resistant to teicoplanin (64 mg/L). According to the Spanish studies, 0.55 % of CoNS strains exhibiting either decreased levels of susceptibility or true resistance to teicoplanin [9].

Two (4%) out of 48 HCWs hands were colonized with two types of strains, *S. aureus *4503, which was the only intermediate resistant to teicoplanin (MIC 16 mg/L) as well as *S. haemolyticus *4509. The last strain was an important strain because vancomycin MIC for its subpopulations was closely to intermediate (7 mg/L) and also resistant to teicoplanin. The role of these two HCW for spreading of the strains was not investigated.

The treatment failures due to the selection of teicoplanin resistant mutants have been reported following treatment of *S. aureus *infections with teicoplanin [12,13,25]. A study made by Wong et al [26], showed that the patients infected by heteroresistant strains have higher mortality rates than the patients infected by sensitive isolates. Other studies on treatment failure associated with heterogeneous VISA have been reported as well [1,5,27]. Our three infected patients with hetero-CoNS have been treated successfuly with vancomycin, on the other hand, death of the third patient was not related to the infection (Table 1).

Although the number of *S. aureus and *CoNS strains included in this study were low, and it is not enough to predict the prevalence. This first study from one of the four largest university hospitals from Turkey may give an idea regarding the status of resistance to glycopeptide among *Staphylococcus *strains, and as a preliminary study need to be followed up with other studies.

## Conclusions

Our study showed that CoNS strains have an emerging heteroresistance for both vancomycin and teicoplanin more than those of *S. aureus *strains and this difference was found to be significant (P = 0.001, P = 0.007 for vancomycin and teicoplanin, respectively). We suggested to screen clinical *S. aureus *and CoNS strains, systematically, in case of the presence of heterogeneously resistance to glycopeptide to avoid treatment failure or any nosocomial outbreaks in the future.

## Competing interests

The author(s) declare that they have no competing interests.

## Authors' contributions

YN participated in the study design and was principal writer of the manuscript. YN, SD, and HK carried out the laboratory studies; AC was responsible for collecting the data from patients medical records and treatment of the patients.

## Pre-publication history

The pre-publication history for this paper can be accessed here:


