# Correction: Investigation of Staphylococcus strains with heterogeneous resistance to glycopeptides in a Turkish university hospital

**DOI:** 10.1186/1471-2334-5-65

**Published:** 2005-08-19

**Authors:** Yasar Nakipoglu, Sengul Derbentli, Atahan A Cagatay, Handan Katranci

**Affiliations:** 1 Department of Microbiology and Clinical Microbiology, Faculty of Medicine, University of Istanbul, Istanbul, Turkey; 2 Department of Infectious Diseases and Clinical Microbiology, Faculty of Medicine, University of Istanbul, Istanbul, Turkey

## Text

We feel that it is our ethical responsibility to add the following acknowledgment to the article titled "Investigation of Staphylococcus strains with heterogeneous resistance to glycopeptides in a Turkish university' [1]:

'We greatly thank Dr. Keichii Hiramatsu and Dr. Maria Kapi from Juntendo University, Tokyo, Japan for their collaboration in confirming of the population analysis.

## Pre-publication history

The pre-publication history for this paper can be accessed here:


